# DTX-P7, a peptide–drug conjugate, is highly effective for non-small cell lung cancer

**DOI:** 10.1186/s13045-022-01274-8

**Published:** 2022-06-03

**Authors:** Yao Jiang, Wei Huang, Xiaojiao Sun, Xiaozhou Yang, Youming Wu, Jiaojiao Shi, Ji Zheng, Shujie Fan, Junya Liu, Jun Wang, Zhen Liang, Nan Yang, Zhenming Liu, Yanyong Liu

**Affiliations:** 1 Department of Pharmacology, Institute of Basic Medical Sciences, Chinese Academy of Medical Sciences & School of Basic Medicine, Peking Union Medical College, Beijing, 100005 China; 2 State Key Laboratory of Natural and Biomimetic Drugs, School of Pharmaceutical Sciences, Peking University, Beijing, 100191 China

**Keywords:** Non-small cell lung cancer, Heat shock protein 90 (Hsp90), Cancer stem-like cells (CSLCs), Cell cycle reentry, Dormancy, Targeting delivery, Unfolded protein response

## Abstract

Despite tremendous success of molecular targeted therapy together with immunotherapy, only a small subset of patients can benefit from them. Chemotherapy remains the mainstay treatment for most of tumors including non-small cell lung cancer (NSCLC); however, non-selective adverse effects on healthy tissues and secondary resistance are the main obstacles. Meanwhile, the quiescent or dormant cancer stem-like cells (CSLCs) are resistant to antimitotic chemoradiotherapy. Complete remission can only be realized when both proliferative cancer cells and quiescent cancer stem cells are targeted. In the present research, we constructed a cooperatively combating conjugate (DTX-P7) composed of docetaxel (DTX) and a heptapeptide (P7), which specifically binds to cell surface Hsp90, and assessed the anti-tumor effects of DTX-P7 on non-small cell lung cancer. DTX-P7 preferentially suppressed tumor growth compared with DTX in vivo with a favorable distribution to tumor tissues and long circulation half-life. Furthermore, we revealed a distinctive mechanism whereby DTX-P7 induced unfolded protein response and eventually promoted apoptosis. More importantly, we found that DTX-P7 promoted cell cycle reentry of slow-proliferating CSLCs and subsequently killed them, exhibiting a “proliferate to kill” pattern. Collecitvely, by force of active targeting delivery of DTX via membrane-bound Hsp90, DTX-P7 induces unfolded protein response and subsequent apoptosis by degrading Hsp90, meanwhile awakens and kills the dormant cancer stem cells. Thus, DTX-P7 deserves further development as a promising anticancer therapeutic for treatment of various membrane-harboring Hsp90 cancer types.

**Graphical Abstract:**

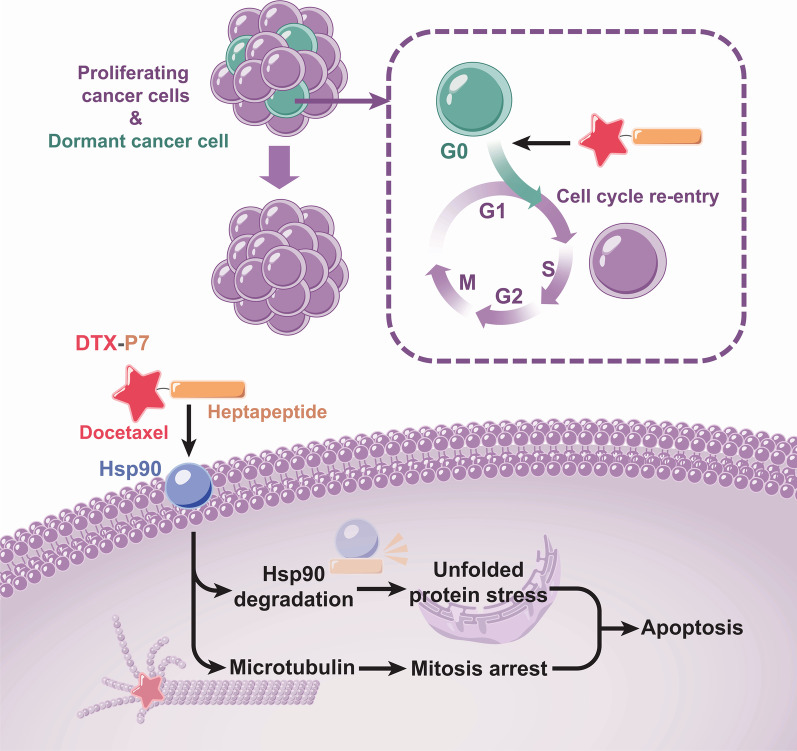

**Supplementary Information:**

The online version contains supplementary material available at 10.1186/s13045-022-01274-8.

**To the editor,**

To address off-target toxicity of conventional chemotherapy, one effort is to enhance the targeted delivery by conjugating therapeutic effector through a cleavable linker to a ligand specific to a drug target, and several conjugates of targeting agents have been approved for application in oncology [1, 2]. However, simply enhancing drug delivery is of limited therapeutic benefit due to insufficiency to overcome the multitude of aberrant cellular processes. Strategies that simultaneously target multiple pathways will bring an opportunity to overcome the common obstacles such as drug resistance. Recently, we successfully identified a heptapeptide (LPLTPLP, namely P7) by phage display technique, which specifically binds to cell surface heat shock protein 90 (Hsp90) and reduces intracellular Hsp90 level in non-small cell lung cancer (NSCLC) cells [3]. It is well known that Hsp90 represents an attractive cancer therapeutic target with unique characteristics whereby its inhibition results in destabilization of multiple signaling pathways [4, 5]. More importantly, the Hsp90α isoform, but not Hsp90β, is expressed on cell surface where it is involved in tumor invasiveness [6–10]. Therefore, it may be feasible to construct a peptide–drug conjugate to realize multifunctional effects including targeted delivery, cellular Hsp90 inhibition and combination with conventional drugs.

Herein, we designed and identified a peptide-conjugated drug comprising P7 and docetaxel (DTX) (namely DTX-P7) (Additional file 2: Fig. S1). We first evaluated the in-vivo efficacy of DTX-P7 in a mouse xenograft model of A549 cells. As shown in Fig. 1a, b, intraperitoneal administration of 20 mg/kg DTX-P7 (equivalent to DTX dose calculated as DTX) reduced tumor growth by 93.2% compared with control mice, whereas the tumor growth inhibition of DTX was only 35.9%. To investigate the targeting characteristics of DTX-P7, the biodistribution of both DTX and DTX-P7 was analyzed by high-performance liquid chromatography. DTX-P7 exhibited active targeting property by preferentially distributing to the tumor tissues (Fig. 1c and Additional file 3: Fig. S2).

**Fig. 1:**
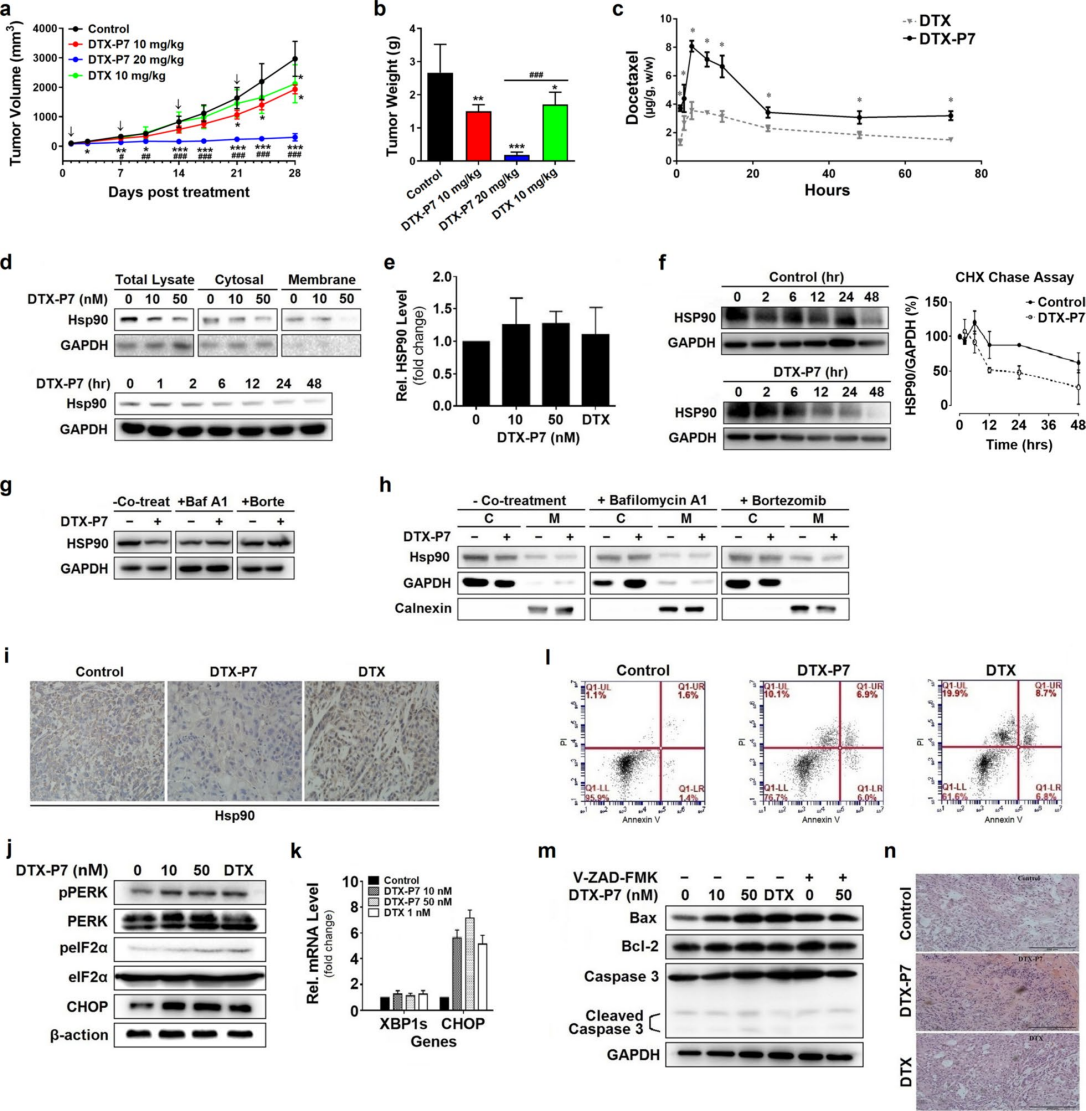
DTX-P7 inhibits tumor growth and promotes tumor cells to apoptosis by favorably distributing to tumor tissues and inducing Hsp90 degradation and unfolded protein response. **a** Volume of xenograft tumor mass. A549 tumor-bearing mice were randomized to receive intraperitoneal injection of vehicle control, 10 mg/kg DTX-P7, 20 mg/kg DTX-P7 or 10 mg/kg DTX once a week for 4 weeks (*n* = 5 mice/group). Data are represented by mean ± SD. Arrows indicate the treatments. **b** Weight of finally dissected xenograft tumor mass. **c** A549 tumor-bearing mice were injected intraperitoneally with 30 mg/kg DTX or 60 mg/kg DTX-P7 followed by determination of distribution of DTX or DTX-P7 in tumor specimens throughout 72 h. DTX-P7 was quantified by free DTX released from the conjugate. **d** A549 cells were treated with different concentrations of DTX-P7 or 50 nM DTX-P7 for different intervals followed by total cell lysate preparation and Western blotting analysis of Hsp90 expression. **e** A549 cells were treated with 0, 10, 50 nM DTX-P7 or 1 nM DTX for 48 h followed by total RNA extraction and real-time PCR for analysis of Hsp90 mRNA level. **f** A549 cells were treated with cycloheximide (2.5 mg/mL) in the presence or absence of 50 nM DTX-P7 for various times and harvested for Western blotting analysis. Half-life of Hsp90 was determined using Image J software and plotted against treatment time. Data shown are mean ± standard deviation of three independent experiments. **g–h** A549 cells were treated with 50 nM DTX-P7 in the absence or presence of 10 nM bafilomycin A_1_ or 20 nM bortezomib for 48 h and harvested for Western blotting analysis of Hsp90 expression in total cell lysates (**g**), cytosol fraction and membrane fraction (**h**). GAPDH and calnexin were used as loading controls for cytosol and membrane fractions, respectively. C: cytosol fraction; M: membrane fraction. **i** Immunohistochemistry analysis of xenograft tumor tissues for the expression of Hsp90. **j** Effect of DTX-P7 on unfolded protein response-related proteins in A549 cells as determined by Western blotting and the quantitative analysis of blots. β-actin was used as a loading control. **k** Effect of DTX-P7 on XBP1 splicing and CHOP mRNA level in A549 cells as determined by real-time PCR analysis. GAPDH was used as an internal control. **l** Effect of DTX-P7 on apoptosis in A549 cells by Annexin V-PI apoptotic assay. **m** A549 cells were incubated with 0, 10, 50 nM DTX-P7 or 1 nM DTX in the presence and absence of Z-VAD-FMK. Total cell lysates were subjected to Western blotting to assess apoptotic proteins using specific antibodies. **n** Hematoxylin and eosin-stained paraffin sections of tumor tissues of vehicle-, DTX- and DTX-P7-treated mice. Scale bar indicated 200 µm. *p < 0.05, **p < 0.01, ***p < 0.001 versus control group; ^#^*p* < 0.05, ^##^*p* < 0.01, ^###^*p* < 0.001 versus DTX group

Our previous study has shown that the heptapeptide P7 specifically binds to cell surface Hsp90 and inhibits expression of intracellular Hsp90 [3]. In this study, DTX-P7 inhibited the expression of membrane Hsp90 in dose- and time-dependent manners, while no change in mRNA level was observed, indicating posttranscriptional regulation of DTX-P7 (Fig. 1d, e). Cycloheximide chase assay and co-treatment of cells with bafilomycin A_1_ or bortezomib verified that DTX-P7 accelerates the degradation of Hsp90 through lysosome- and proteasome-dependent pathways (Fig. 1f–h). Furthermore, immunohistochemistry staining analysis of xenograft tumors also revealed decreased Hsp90 level in DTX-P7-treated mice, whereas treatment with DTX induced a significant increase in Hsp90 expression, reflecting an active adaption of tumor (Fig. 1i).

To identify the downstream molecular mechanism of DTX-P7-induced Hsp90 degradation, we performed a label-free quantitative proteomic analysis. The data demonstrated a widespread impact of DTX-P7 on proteins in cells and provided insight into the cellular pathways associated with phagosome, protein processing in endoplasmic reticulum (ER) and PI3K-Akt signaling pathway (Additional file 4: Fig. S3). We then confirmed the change of protein processing in endoplasmic reticulum by Western blotting analysis. As shown in Fig. 1j, k, DTX-P7 enhanced phosphorylation of PERK and eIF2α, whereas no XBP-1 RNA splicing was observed when A549 cells were exposed to DTX-P7, suggesting that DTX-P7 treatment induces the unfolded protein response (UPR) via PERK/eIF2α pathway. When the adaptive mechanism fails to restore normal ER function due to protracted or excessive stress stimuli, the UPR pathways may initiate apoptotic pathways to remove the stressed cells [11]. For this reason, we observed cooperative combating effect of DTX-P7 to promote cell apoptosis (Fig. 1l–n). Based on the above findings, we conclude that DTX-P7 induces tumor cells to apoptosis and eventually death.

Cytotoxic agents that target killing rapidly proliferating cells are often difficult to produce good curative effects in the dormant or quiescent cancer stem cells in tumor tissues [12]. The presence of Hsp90 protein on the membrane of A549/CD133^+^ cancer stem cells was first confirmed, highlighting the potential application of P7 (Additional file 5: Fig. S4). Despite insignificant difference in vitro, DTX-P7 significantly reduced tumor growth in mice bearing xenograft of A549/CD133^+^ cells (Additional file 6: Fig. S5a and Fig. 2a, b). By staining with PKH26, a lipophilic dye which declines with every round of division and distinguishes rapidly and slowly proliferating cells by fluorescence intensity in cells, we found that both quiescent/slowly proliferating cells and fast proliferating cells were more sensitive to DTX-P7 than DTX (Fig. 2c and Additional file 6: Fig. S5b, c). In addition, DTX-P7 significantly reduced G_0_/G_1_ cells and arrested them in G_2_/M phase (Fig. 2d, e). DTX-P7 also reduced the expression of DYRK1A, which is known to regulate G_1_ phase where the cell cycle entry versus exit decision is made (Fig. 2f, g). A rise in cyclin D1 and a decline in p21 were observed as well. As a client protein of Hsp90, DYRK1A showed obvious ubiquitination following DTX-P7 treatment in A549/CD133^+^ cells (Fig. 2h). All these findings suggest that DTX-P7 suppresses survival of quiescent/slowly proliferating A549/CD133^+^ cells via degradation of DYRK1A and subsequent cell cycle reentry.

**Fig. 2:**
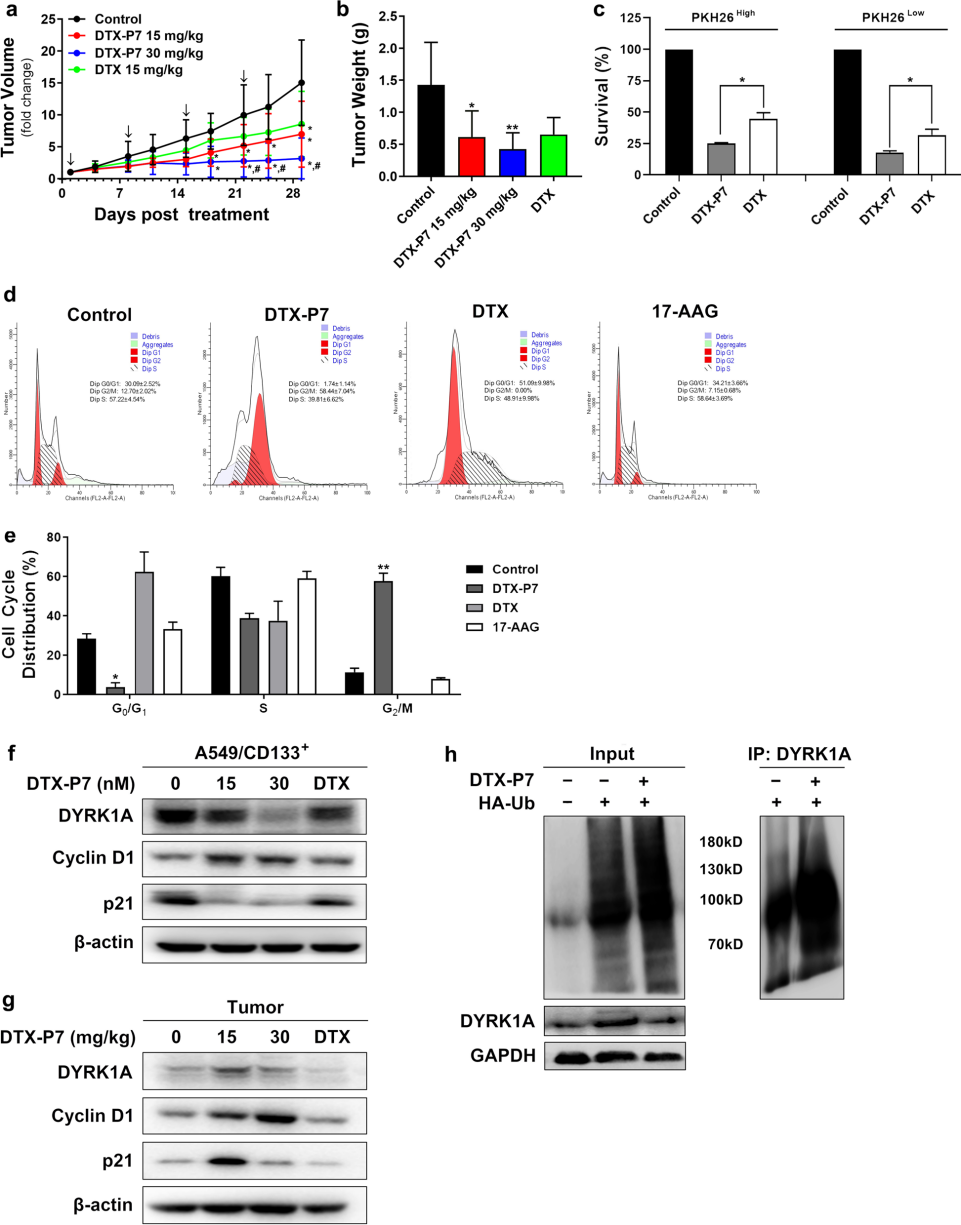
DTX-P7 suppresses tumor growth of quiescent/slowly proliferating A549/CD133^+^ cells via degradation of DYRK1A and subsequent cell cycle reentry. **a** Volume of xenograft tumor mass. A549/CD133^+^ tumor-bearing mice were randomized into receive intraperitoneal injection of vehicle control, 15 mg/kg DTX-P7, 30 mg/kg DTX-P7 or 15 mg/kg DTX once a week for 4 weeks (*n* = 7 mice/group). Data are represented by mean ± SEM. Arrows indicate the treatments. **b** Tumor tissues were dissected and weighed when the animals were euthanized in 4 weeks post treatment. **c** A549/CD133^+^ mock-sorted (untreated) or sorted into PKH26^high^ and PKH26^low^ populations were incubated for 72 h with 10 μM DTX or 10 μM DTX-P7 followed by cell viability assay. **d**–**e** A549/CD133^+^ cells were treated with vehicle control, DTX-P7, DTX or 17-AAG for 48 h followed by cell cycle analyses by propidium iodide. Panel e represents three independent experiments. **f** A549/CD133^+^ cells were treated with 0, 15, 30 nM DTX-P7 or 15 nM DTX for 48 h followed by total cell lysate preparation and Western blotting analysis. β-actin was used as a loading control. **g** Total lysate preparation and Western blotting analysis of tumor tissues. β-actin was used as a loading control. **h** DYRK1A was reduced by ubiquitination. A549/CD133^+^ cells were transfected with HA-Ubiquitin followed by treatment with vehicle control or 30 nM DTX-P7 for 24 h. The lysates were immunoprecipitated using an anti-DYRK1A antibody, followed by immunoblotting with an anti-HA antibody under denaturing conditions. *p < 0.05, **p < 0.01, versus control group

Collectively, a novel multifunctional DTX-P7 was successfully constructed for cancer therapy. In addition to active targeting delivery of DTX via Hsp90, DTX-P7 induces unfolded protein response and subsequent apoptosis, and awakens and kills the dormant cancer stem cells, making it a promising targeting strategy for lung cancer and other cancers where Hsp90 is highly expressed on cell surface.

## Supplementary Information

**Additional file 1:** Methods.

**Additional file 2: Supplementary Figure S1.** Characterization of DTX-P7 conjugate. a) Chemical structures of DTX and DTX-P7. DTX, molecular formula: C_43_H_53_NO_14_, molecular weight: 807.9 g/mol, white powder. DTX-P7, molecular formula: C_84_H_118_N_8_O_25_, molecular weight: 1,639.90 g/mol, white powder. b-c) Cell viability of DTX-P7 and DTX in A549 (b) and H1975 (c) cells following 48-h treatment. IC_50_ values: A549 cells, DTX-P7 11.4 nM, DTX 1.11 nM; H1975 cells, DTX-P7 0.62 nM, DTX 0.50 nM.

**Additional file 3: Supplementary Figure S2.** Biodistribution of DTX-P7 in nude mice bearing A549 xenograft tumor. DTX-P7 was quantified by free DTX released from the conjugate. a) Plasma concentration of DTX and DTX-P7 in plasma samples throughout 72-h treatment. b-e) Distribution of DTX in the heart, liver, spleen, lungs, kidneys, and brain in 1 h (b), 2 h (c), 4 h (d) and 8 h (e) after DTX or DTX-P7 was administrated to mice implanted with A549 xenograft tumor. Data are given as mean ± SD (*n* = 3). * *p* < 0.05 vs. DTX group.

**Additional file 4: Supplementary Figure S3.** Proteome changes induced in A549 cells by DTX-P7. a-c) Gene Ontology (GO) annotation analysis of A549 cell proteins that changed more than 1.5-ratio after DTX-P7 treatment, including altered proteins for biological process (a), cellular component (b) and molecular function (c) analyses. d) Kyoto Encyclopedia of Genes and Genomes (KEGG) pathway annotation analysis of A549 cell proteins that changed more than 1.5-ratio after DTX-P7 treatment.

**Additional file 5: Supplementary Figure S4.** Cell growth morphology of cancer stem cell-like A549/CD133^+^ cells and identification of cell surface Hsp90 in A549/CD133^+^ cells. a) Morphology of A549 and A549/CD133^+^ cells. b) Hsp90 expression levels were assessed in A549/CD133^+^ cells by cellular fractionation and Western blotting analysis. c) Immunofluorescence analysis of cell surface Hsp90 in A549/CD133^+^ cells. d) Immunofluorescence assays of FITC-labeled P7 binding to A549/CD133^+^ cells. Competition of P7 binding to A549/CD133^+^ cells by excess free P7.

**Additional file 6: Supplementary Figure S5.** Effects of DTX-P7 on survival of A549/CD133^+^ cells and PKH26 staining of A549/CD133^+^ cells. a) Cell viability of DTX-P7 and DTX in A549/CD133^+^ cells following 48-h treatment. b) A549/CD133^+^ cells were stained by PKH26 followed by fluorescence detection at Day 0, 1, 6 and 10. c) Representative fluorescence-activated cell sorting profile of A549/CD133^+^ cells selected for sorting 10 days after PKH26 staining as compared with those of the unstained control (negative control) and Day 0.

## Data Availability

All data generated or analyzed during this study are included in this published article and its supplemental material file.

## Abbreviations

CSLC: Cancer stem-like cell
DTX: Docetaxel
DYRK1A: Dual-specificity tyrosine-(Y)-phosphorylation-regulated protein kinase 1A
eIF2α: Eukaryotic translation initiation factor 2α
ER: Endoplasmic reticulum
Hsp90: Heat shock protein 90
NSCLC: Non-small cell lung cancer
PERK: Protein kinase R-like endoplasmic reticulum kinase
PI3K: Phosphatidylinositol 3-kinase
UPR: Unfolded protein response
XBP-1: X-box-binding protein-1
