# Supplementary material for: DTX-P7, a peptide–drug conjugate, is highly effective for non-small cell lung cancer
Source: J Hematol Oncol. 2022 Jun 3;15:73. doi: 10.1186/s13045-022-01274-8 (PMC9164557; doi:10.1186/s13045-022-01274-8)
Supplement: Supplementary file 1 — Additional file 1: Methods. [file 13045_2022_1274_MOESM1_ESM.pdf]

Supplemental Material

DTX-P7, a peptide-drug conjugate, is highly effective for non-small cell lung cancer

Methods

Chemicals and reagents

The heptapeptide P7 and FITC-labeled P7 were purchased from Chinapeptides Co., Ltd. (Shanghai, China). DTX was purchased from the Beijing Zhongshuo Pharmaceutical Technology Development Co., Ltd. (Beijing, China). DTX-P7 was synthesized by Shenzhen Keyshine Trading Co., Ltd. (Shenzhen, Guangdong, China) and purified via high performance liquid chromatography (HPLC) with a purity of > 98%. All the other reagents of analytical grade were purchased from the Sinopharm Chemical Reagent Co., Ltd (Beijing, China). Antibodies against Hsp90 (Cat. No. 4877), p-PERK (Cat. No. 3179), PERK (Cat. No. 5683), p-eIF2α (Cat. No. 3398), eIF2α (Cat. No. 5324), CHOP (Cat. No. 2895), Bax (Cat. No. 2772), Bcl-2 (Cat. No. 2870), Caspase 3 (Cat. No. 9662), cleaved-Caspase 3 (Cat. No. 9664), DYRK1A (Cat. No. 8765), Cyclin D1 (Cat. No. 2978), p21 (Cat. No. 2947), β-actin (Cat. No. 4970) and HA tag (Cat. No. 3724) were purchased from Cell Signaling Technology (Danvers, MA, USA). Antibody against GAPDH (Cat. No. sc-25778) was purchased from Santa Cruz Biotechnology (Santa Cruz, CA, USA). All antibodies were diluted as recommended by the manufacturer's instructions unless otherwise specified.

Cell culture and cell viability assay

Human non-small cell lung cancer cells A549 and H1975 were obtained from the Cell Bank of Institute of Basic Medical Sciences, Chinese Academy of Medical Sciences (Beijing, China), and

cultured in RPMI 1640 medium (Macgene, Beijing, China) containing 10% fetal bovine serum (FBS) (PAN-Biotech, GmbH, Aidenbach, Germany) and 1% penicillin/streptomycin (Macgene) in a 5% CO_2 incubator at 37°C. A549/CD133$^+$ cells were cultured in DMEM/F-12 medium (Macgene) supplemented with B27 (Invitrogen, Carlsbad, CA, USA), 20 ng/ml EGF (PeproTech, Rochy Hill, NJ, USA), 20 ng/ml bFGF (PeproTech) and appropriate antibiotics in a 5% CO_2 incubator at 37°C as described previously (1). Cytotoxicity of DTX-P7 and DTX was evaluated utilizing Cell Counting Kit-8 (Dojindo Laboratories, Kumamoto, Japan) as we previously described (2).

Evaluation of anti-tumor efficacy in xenograft mouse model

BALB/c nude mice were purchased from Beijing Weitong Lihua Animal Center (Beijing, China). Animal research was approved by the Center for Experimental Animal Research of Institute of Basic Medical Sciences, Chinese Academy of Medical Sciences. The efficacy of DTX-P7 was examined in male BALB/c nude mice (20-25 g) subcutaneously injected with 2×10^6 A549 cells. When tumors had reached >100 mm^3 in size, mice were randomized into receive intraperitoneal injection of vehicle control (10% castor oil: ethanol (1:1) and 90% normal saline), 10 mg/kg DTX-P7, 20 mg/kg DTX-P7 or 10 mg/kg DTX once a week for 4 weeks (n = 5 mice/group). Tumor volume was measured with calipers and calculated as (length×width^2)/2. To evaluate the histological alternation, paraffin-embedded tissue sections were stained with hematoxylin and eosin (H&E) and then evaluated by a pathologist who was blind to the present experiment. For mouse xenograft model of A549/CD133$^+$ cells, 1×10^5 A549/CD133$^+$ cells were resuspended in a mixture of PBS and Matrigel (1:1) and subcutaneously injected into the flank of BALB/c nude mice (6-8 weeks). When the tumor volume grew up to >100 mm^3, mice were randomly divided into vehicle control group, 15 mg/kg DTX group, 15 mg/kg DTX-P7 group and 30 mg/kg DTX-P7 group with intraperitoneal injection once a week for 4 weeks. Tumor volume and weight were measured every two to three days.

Pharmacokinetic and tissue biodistribution analyses

BALB/c mice bearing A549 tumors (~100 mm^3) were injected intraperitoneally with 30 mg/kg DTX or 60 mg/kg DTX-P7. Compared with the dosage for pharmacodynamics, a higher dosage was adopted here to reveal the comprehensive pharmacokinetic profiles of DTX-P7. 1, 2, 4, 12, 24, 48 and 72 h after injection, the mice were anesthetized, and approximately 0.5 ml of blood was collected by cardiac puncture into anticoagulant tubes and centrifuged at 1,000 ×g for 10 min to obtain the plasma. At each time point, the tumor, brain, heart, liver, spleen, lungs, and kidneys were dissected and weighed. The plasma and tissues were stored at -20°C. To quantify DTX and DTX-P7 in the tissues, chromatography was performed on a HPLC system (Waters, Model 2960) equipped with C_{18} column (150 nm × 4.6 mm, 5 μm). The flow rate of mobile phase (acetonitrile-water (45:55) - 0.1% trifluoroacetic acid) was 1.0 ml/min. Peaks were detected by Waters 996 photodiode array detector at the wavelength of 230 nm. The column temperature was 24±1°C. Standard calibration curve was obtained by adding known amounts of DTX-P7/DTX to drug-free plasma. The concentrations of DTX-P7 were calculated by the peak area.

Label-free liquid chromatography-mass spectrometry for quantitative proteomics

Protein extraction, sample preparation and proteomic analysis were performed by Beijing BangFei Bioscience Co., Ltd. (Beijing, China). Briefly, cell pellets were lysed with lysis buffer (8 M urea, 50 mM NH_4HCO_3, 1× protease inhibitor) followed by brief sonication and centrifugation at 13,000 rpm for 5 min at 4°C. 60 μg of protein sample was incubated with 5 μL of 1 M DTT at 37°C for 1 h and then 20 μL of 1 M IAA solution at room temperature for 1 h, protected from light. Following ultracentrifugation once, 100 μL of 8 M urea in 0.1 M Tris·HCl, pH 8.5 were added to the ultrafiltrate with two repetitive steps, and 100 μL of 50 mM NH_4HCO_3 were then added to the ultrafiltrate with three repetitive steps. Digestion with trypsin was carried out at a ratio of 1:50 at 37°C overnight. Each digested-peptide sample was automatically loaded onto a C_{18} trap column (3 μm 0.10×20 mm), which was connected to a C_{18} separation column (1.9 μm 0.15×120 mm),

with two solvent buffers (A: 99.9% water and 0.1% formic acid; B: 80% acetonitrile, 19.92% water, and 0.08% formic acid) for 75 min gradient of acetonitrile at a flow rate of 600 nl/min. Peptides were separated on Dionex Ultimate 3000 HPLC system (Thermo Scientific, Waltham, MA, USA) and analyzed by using Q Exactive^{TM} HF hybrid quadrupole-orbitrap mass spectrometer (Thermo Scientific) with a resolution of 120, 000 at a full scan mode and 15, 000 at the MS/MS mode. Mascot and Proteome Discoverer (Thermo Scientific) were used for data analysis.

Flow cytometric analysis of apoptosis and cell cycle

To quantify apoptosis, the treated cells were collected and stained with Annexin V-FITC and PI by using Annexin V/PI Apoptosis Detection Kit (Dojindo Laboratories) according to the supplier's instructions as we previously described (3). Apoptotic cells were finally analyzed by BD Accuri^{TM} C6 Flow Cytometer (BD Biosciences, San Jose, CA, USA). For cell cycle analysis, approximately 1×10^6 cells were harvested, washed twice with ice-cold PBS, and fixed in 70% ethanol overnight. Then, cells were stained with 0.5 ml of PBS containing propidium iodide. Flow cytometry was performed on BD Accuri^{TM} C6 Flow Cytometer to detect cell cycle distribution.

PKH26 staining

A549/CD133$^+$ spheroids were dissociated with StemPro^{TM} Accutase^{TM} Cell Dissociation Reagent (Invitrogen) and stained for 1 h at 37°C with 1:500 PKH26 (Sigma-Aldrich, St. Louis, MO, USA). After extensive washing with PBS, cells were cultured for additional 10 days and sorted with BD FACSAria^{TM} II flow cytometry system (BD Biosciences, San Jose, CA, USA) into PKH^{low} and PKH^{high} fractions. Unstained control cells were mock-sorted to induce similar stress conditions as in the sorted samples.

Fluorescence microscopy

To evaluate autophagic activity, LC3 translocation was detected using the green fluorescent protein (GFP)-fused LC3 construct. Briefly, A549 cells were transfected with LC3-GFP plasmid using Lipofectamine 2000 (Invitrogen). 24 h later, cells were treated with DTX-P7 or DTX. Fluorescence analysis was performed on an Olympus DP72 microscope.

Western blotting

Cells were collected and lysed in ice-cold TNN buffer (50 mM Tris·HCl, pH 7.4, 150 mM NaCl, 20 mM EDTA, 0.5% NP-40, 1 mM Na_3VO_4, 50 mM NaF) with 1 mM PMSF and 1 mM DTT. After 10-min incubation on ice and brief sonication, the lysates were centrifuged at 12,000 ×g for 20 min at 4°C to collect the supernatants. Protein concentrations of cell lysates were determined by Bicinchoninic Acid Protein Assay Kit (Dingguo Changsheng Biotechnology, Beijing, China). For western blot analysis, equal amounts of lysates were separated by 8~15% sodium dodecyl sulfate polyacrylamide gel electrophoresis and transferred to polyvinylidene fluoride membranes (EMD Millipore, Billerica, MA, USA) that were probed with the appropriate primary antibodies at 4°C overnight, followed by the corresponding secondary antibodies for 1 h at room temperature. Immunoactivity was detected and visualized by an enhanced chemiluminescence.

Real-time polymerase chain reaction (PCR)

Real-time qPCR was performed to determine the mRNA expression levels as we previously described (4). Total RNA was isolated from cells using E.Z.N.A.^{TM} Total RNA Kit I (Omega Bio-tek, Norcross, GA, USA) followed by reverse transcription to cDNA using All-In-One RT MasterMix Kit (Applied Biological Materials, Richmond, BC, Canada). The resultant cDNA was subjected to 95°C for 10 min and 40 cycles of PCR amplification on StepOnePlus PCR system (Applied Biosystems, Waltham, MA, USA) using EvaGreen qPCR MaxterMix (Applied Biological Materials) under the following cycling condition: 95°C for 15 sec and 60°C for 1 min. The primers for the genes of interest were shown as follows: CHOP: forward, 5'-

AGCGACAGAGCCAAAATCAG-3’, reverse, 5’-ACAAGTTGGCAAGCTGGTCT-3’; Hsp90: forward, 5’-GCTTATTTGGTTGCTGAGAAAGTAACT-3’, reverse, 5’-TTCCACGACCCATAGGTTCAC-3’; GAPDH: forward, 5’-AAGGACTCATGACCACAGTCCAT-3’, reverse, 5’-CCATCACGCCACAGTTTCC-3’; XBP1s: forward, 5’-TGCTGAGTCCGCAGCAGGTG-3’, reverse, 5’-GCTGGCAGGCTCTGGGGAAG-3’. GAPDH was used as the internal control. The relative mRNA levels were determined using $2^{-\Delta\Delta Ct}$ method.

Immunofluorescence staining

After being washed three times with ice-cold PBS, the cells were fixed with paraformaldehyde (4%) for 30 min, washed three times with PBS, and blocked with 10% FBS in PBS for 30 min at room temperature. The cells were then incubated with anti-Hsp90 antibody diluted 1:50 (v./v.) in PBS overnight at 4°C, followed by incubation with FITC-conjugated goat anti-rabbit IgG (ZSGB Biotech, Beijing, China) at room temperature for 1 h. For P7 targeting detection, after fixing in paraformaldehyde, washing with PBS, and blocking with 10% FBS in PBS, the cells were assigned to be incubated with FITC control, FITC-labelled P7 or unlabeled P7 for 1 h at room temperature. These coverslips were then washed thrice with PBS and incubated with 4’, 6-diamidine-2-phenylindole dihydrochloride (DAPI) (300 nM in PBS) for 30 min in the dark. The coverslips were then washed thrice with distilled water and mounted on slides before viewing with an Olympus DP72 microscope.

Ubiquitination assay

HA-ubiquitin (HA-Ub) construct was a generous gift from Dr. Deng Chen at Institute of Basic Medical Sciences, Chinese Academy of Medical Sciences (5). A549/CD133^{+} cells were transfected with 6 μg of HA-Ub plasmid with VigoFect transfection reagent (VigoFect, Beijing, China). 8 h later, cells were replaced with fresh medium and treated with vehicle control or 30 nM

DTX-P7 for 24 h. Cells were then directly harvested and lysed in TNN lysis buffer with 1 mM PMSF, then the cell lysates were passed through 1-ml syringe with 26G needle 5 times and centrifuged at 13,000 rpm for 15 min at 4℃. The supernatants were collected and quantified for protein content by the Bicinchoninic Acid Protein Assay Kit (Dingguo Changsheng Biotechnology). Cell lysates were pre-cleared with IgG and subsequently incubated with anti-DYRK1A antibody (Cell Signaling Technology, Danvers, MA, USA) overnight at 4℃ following addition of 30 μl of magnetic beads in each sample and incubation for 1.5 h at 4℃. Finally, poly-ubiquitination of DYRK1A was analyzed by Western blotting after 3 washings with lysis buffer and denaturing at 95℃ for 5 min.

Statistical analysis

Data were presented as mean ± standard deviation. Statistical differences between two groups were compared using the Student's t test. Multiple comparisons were conducted by one-way analysis of variance (ANOVA) followed by LSD or Dunnett's T3 *post-hoc* test using SPSS ver.18.0. Statistical significance was considered at $p < 0.05$.

References

1. Zhang H, Yang N, Sun B, Jiang Y, Hou C, Ji C, *et al*. CD133 positive cells isolated from A549 cell line exhibited high liver metastatic potential. Neoplasma. 2014; 61(2): 153-60. https://doi.org/10.4149/neo_2014_021
2. Cao M, Huang W, Chen Y, Li G, Liu N, Wu Y, *et al*. Chronic restraint stress promotes the mobilization and recruitment of myeloid-derived suppressor cells through β-adrenergic-activated CXCL5-CXCR2-Erk signaling cascades. Int J Cancer. 2021; 149(2): 460-72. https://doi.org/10.1002/ijc.33552
3. Huang W, Liu Y, Wang J, Yuan X, Jin HW, Zhang LR, *et al*. Small-molecule compounds targeting the STAT3 DNA-binding domain suppress survival of cisplatin-resistant human ovarian cancer cells by inducing apoptosis. Eur J Med Chem. 2018; 157: 887-97. https://doi.org/10.1016/j.ejmech.2018.08.037
4. Huang W, Yuan X, Sun T, Fan SJ, Wang J, Zhou Q, *et al*. Proteasome Inhibitor YSY01A abrogates constitutive STAT3 signaling via down-regulation of Gp130 and JAK2 in human A549 lung cancer cells. Front Pharmacol. 2017; 8: 476. https://doi.org/10.3389/fphar.2017.00476
5. Ma W, Zhang X, Li M, Ma X, Huang B, Chen H, *et al*. Proapoptotic RYBP interacts with FANK1 and induces tumor cell apoptosis through the AP-1 signaling pathway. Cell Signal. 2016; 28(8): 779-87. https://doi.org/10.1016/j.cellsig.2016.03.012
