# Supplementary material for: DTX-P7, a peptide–drug conjugate, is highly effective for non-small cell lung cancer
Source: J Hematol Oncol. 2022 Jun 3;15:73. doi: 10.1186/s13045-022-01274-8 (PMC9164557; doi:10.1186/s13045-022-01274-8)
Supplement: Supplementary file 2 — Additional file 2: Supplementary Figure S1. Characterization of DTX-P7 conjugate. a) Chemical structures of DTX and DTX-P7. DTX, molecular formula: C43H53NO14, molecular weight: 807.9 g/mol, white powder. DTX-P7, molecular formula: C84H118N8O25, molecular weight: 1,639.90 g/mol, white powder. b-c) Cell viability of DTX-P7 and DTX in A549 (b) and H1975 (c) cells following 48-h treatment. IC50 values: A549 cells, DTX-P7 11.4 nM, DTX 1.11 nM; H1975 cells, DTX-P7 0.62 nM, DTX 0.50 nM. [file 13045_2022_1274_MOESM2_ESM.pdf]

Supplemental Material

DTX-P7, a peptide-drug conjugate, is highly effective for non-small cell lung cancer

DTX-P7 suppresses NSCLC cell survival

To determine the effectiveness of DTX-P7 in suppressing cancer cell survival, we performed cytotoxicity assay of the conjugate against lung cancer A549 and H1975 cells. DTX-P7 showed comparable effect with DTX in the two cell lines (supplemental Fig. S1b, c). IC_{50} values of DTX and DTX-P7 for A549 cells were 1.11 nM and 11.4 nM, respectively, while IC_{50} values of DTX and DTX-P7 for H1975 cells were 0.50 nM and 0.62 nM, respectively.

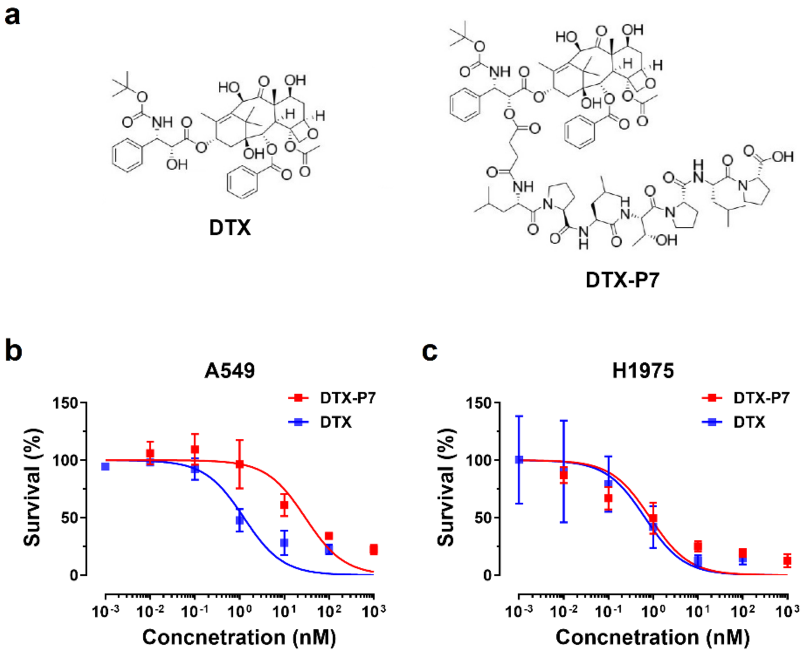

Fig.S1. Characterization of DTX-P7 conjugate. a) Chemical structures of DTX and DTX-P7. DTX, molecular formula: $C_{43}H_{53}NO_{14}$, molecular weight: 807.9 g/mol, white powder. DTX-P7, molecular formula: $C_{84}H_{118}N_8O_{25}$, molecular weight: 1,639.90 g/mol, white powder. **b-c)** Cell viability of DTX-P7 and DTX in A549 (b) and H1975 (c) cells following 48-h treatment. IC_{50} values: A549 cells, DTX-P7 11.4 nM, DTX 1.11 nM; H1975 cells, DTX-P7 0.62 nM, DTX 0.50 nM.
