# Supplementary material for: DTX-P7, a peptide–drug conjugate, is highly effective for non-small cell lung cancer
Source: J Hematol Oncol. 2022 Jun 3;15:73. doi: 10.1186/s13045-022-01274-8 (PMC9164557; doi:10.1186/s13045-022-01274-8)
Supplement: Supplementary file 3 — Additional file 3: Supplementary Figure S2. Biodistribution of DTX-P7 in nude mice bearing A549 xenograft tumor. DTX-P7 was quantified by free DTX released from the conjugate. a) Plasma concentration of DTX and DTX-P7 in plasma samples throughout 72-h treatment. b-e) Distribution of DTX in the heart, liver, spleen, lungs, kidneys, and brain in 1 h (b), 2 h (c), 4 h (d) and 8 h (e) after DTX or DTX-P7 was administrated to mice implanted with A549 xenograft tumor. Data are given as mean ± SD (n = 3). * p < 0.05 vs. DTX group. [file 13045_2022_1274_MOESM3_ESM.pdf]

Supplemental Material

DTX-P7, a peptide-drug conjugate, is highly effective for non-small cell lung cancer

Biodistribution of DTX-P7

To investigate the targeting characteristics of DTX-P7, the biodistribution of both DTX and DTX-P7 was analyzed by HPLC. The distribution of DTX-P7 in tumor tissues and other organs was quantified by DTX released from DTX-P7. DTX-P7 can be detected in the plasma in the first 24 h post treatments with higher area under curve (AUC) and C_{max} than DTX (supplemental Fig. S2a). In normal tissues, docetaxel was barely detectable in brain in 1, 2, 4 and 8 h after injection (supplemental Fig. S2b-e). In the liver, the docetaxel concentration of DTX group was 5 folds and 3 folds higher than that of DTX-P7 group after 1 and 2 h of injection. Similar distribution was also observed in heart and lungs.

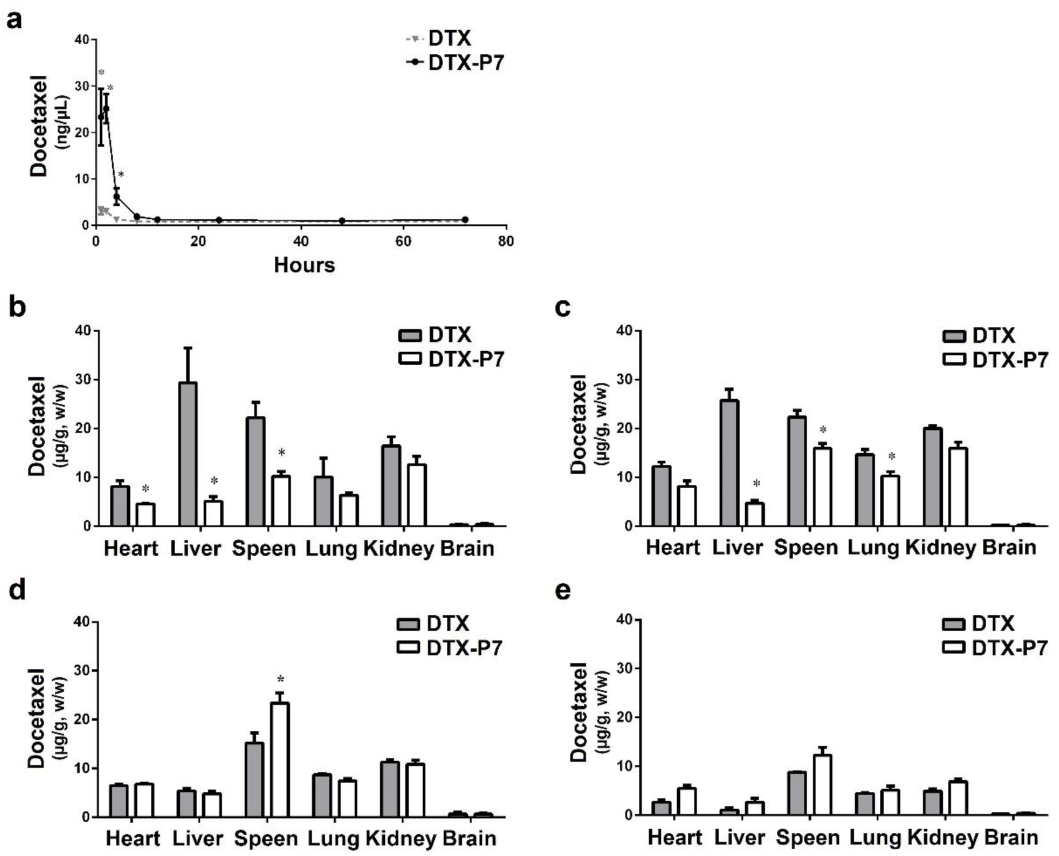

Fig.S2. Biodistribution of DTX-P7 in nude mice bearing A549 xenograft tumor. DTX-P7 was quantified by free DTX released from the conjugate. **a)** Plasma concentration of DTX and DTX-P7 in plasma samples throughout 72-h treatment. **b-e)** Distribution of DTX in the heart, liver, spleen, lungs, kidneys, and brain in 1 h (b), 2 h (c), 4 h (d) and 8 h (e) after DTX or DTX-P7 was administrated to mice implanted with A549 xenograft tumor. Data are given as mean ± SD ($n = 3$). * $p < 0.05$ vs. DTX group.
