# Supplementary material for: DTX-P7, a peptide–drug conjugate, is highly effective for non-small cell lung cancer
Source: J Hematol Oncol. 2022 Jun 3;15:73. doi: 10.1186/s13045-022-01274-8 (PMC9164557; doi:10.1186/s13045-022-01274-8)
Supplement: Supplementary file 4 — Additional file 4: Supplementary Figure S3. Proteome changes induced in A549 cells by DTX-P7. a-c) Gene Ontology (GO) annotation analysis of A549 cell proteins that changed more than 1.5-ratio after DTX-P7 treatment, including altered proteins for biological process (a), cellular component (b) and molecular function (c) analyses. d) Kyoto Encyclopedia of Genes and Genomes (KEGG) pathway annotation analysis of A549 cell proteins that changed more than 1.5-ratio after DTX-P7 treatment. [file 13045_2022_1274_MOESM4_ESM.pdf]

Supplemental Material

DTX-P7, a peptide-drug conjugate, is highly effective for non-small cell lung cancer

Proteome changes induced in A549 cells by DTX-P7

To identify the downstream molecular mechanism of DTX-P7-induced Hsp90 degradation, we performed a label-free quantitative proteomic analysis. In total, 29,753 peptides were identified. The number of proteins identified was 4,234, including 3,693 in control cells, 3,539 following DTX-P7 treatment and 3,665 following DTX treatment. The first step is direct comparison of DTX-P7 treated versus control sample MS peak intensity. Overall, 700 proteins changed, of which 320 increased and 380 decreased after DTX-P7 treatment compared to untreated controls. To characterize the functions and subcellular locations of the differentially expressed proteins in response to DTX-P7 treatment, the differentially expressed proteins were mapped to terms in the Gene Ontology (GO) database (http://www.geneontology.org) with three GO classifications including biological process, cellular component, and molecular function. For the biological process classification in GO annotation, the altered proteins following DTX-P7 treatment were predominantly involved in DNA-templated transcription, neutrophil degranulation, and regulation of transcription from RNA polymerase II promoter (supplemental Fig. S3a). The top three ranks of the cellular components were cytosol, nucleus, and nucleoplasm (supplemental Fig. S3b). The altered proteins involved in ATP binding, RNA binding and metal ion binding were the main proportion of proteins enriched by molecular function analysis (supplemental Fig. S3c). All data suggest a widespread impact of DTX-P7 on proteins in cells. In order to explore the mechanism of DTX-P7, we then focused specifically on the annotation terms linked to KEGG pathway. The Kyoto Encyclopedia of Genes and Genomes (KEGG, http://www.genome.ad.jp/kegg/) database is a knowledge base for systematic analysis of gene junctions. In the present study, the top 30

pathways enriched after DTX-P7 treatment were shown in supplemental Fig. S3d. Our results provided insight into the cellular pathways associated with phagosome, protein processing in endoplasmic reticulum and PI3K-Akt signaling pathway.

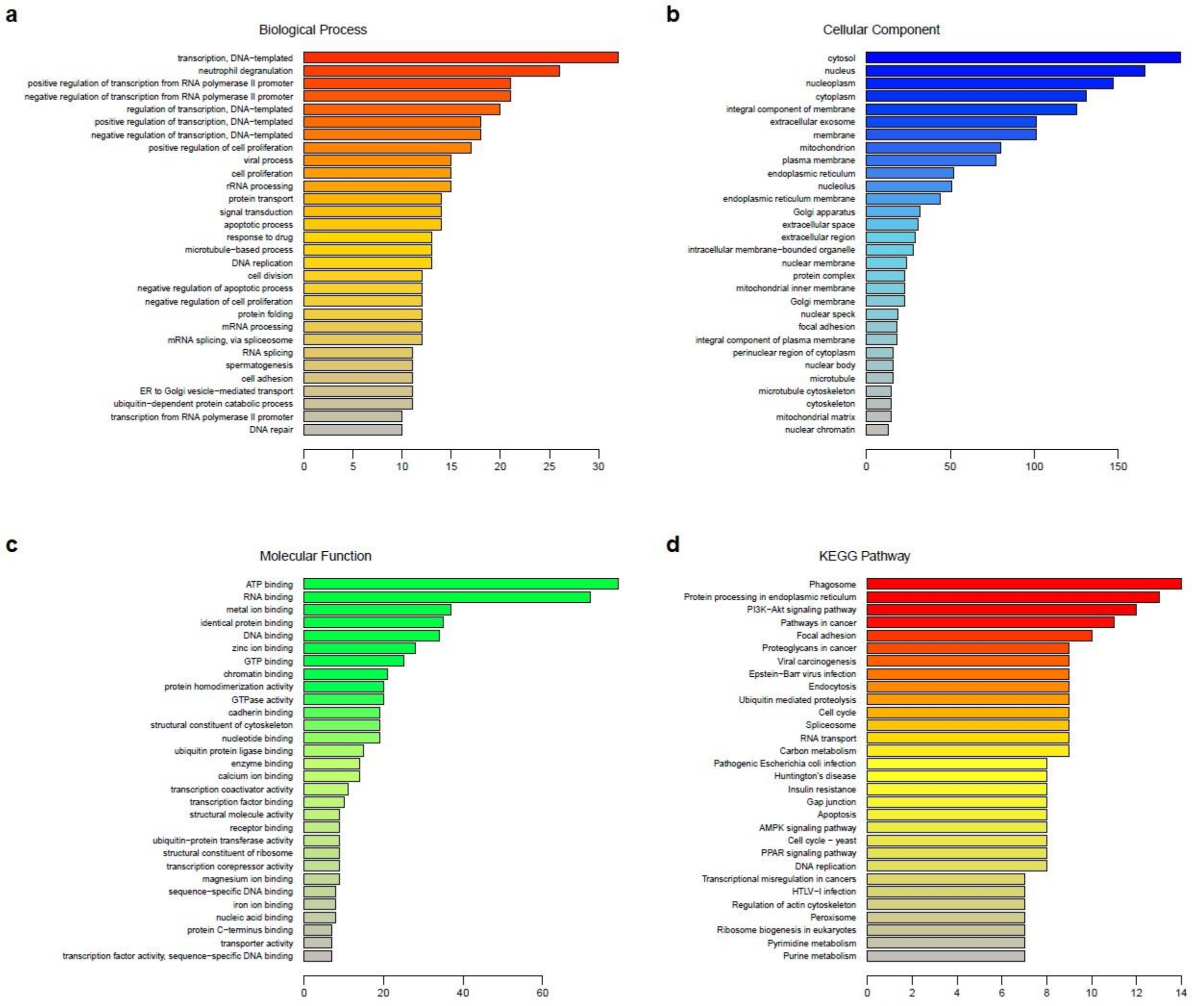

Fig.S3. Proteome changes induced in A549 cells by DTX-P7. a-c) Gene Ontology (GO) annotation analysis of A549 cell proteins that changed more than 1.5-ratio after DTX-P7 treatment, including altered proteins for biological process (a), cellular component (b) and molecular function (c) analyses. **d)** Kyoto Encyclopedia of Genes and Genomes (KEGG) pathway annotation analysis of A549 cell proteins that changed more than 1.5-ratio after DTX-P7 treatment.
