# Supplementary material for: DTX-P7, a peptide–drug conjugate, is highly effective for non-small cell lung cancer
Source: J Hematol Oncol. 2022 Jun 3;15:73. doi: 10.1186/s13045-022-01274-8 (PMC9164557; doi:10.1186/s13045-022-01274-8)
Supplement: Supplementary file 5 — Additional file 5: Supplementary Figure S4. Cell growth morphology of cancer stem cell-like A549/CD133+ cells and identification of cell surface Hsp90 in A549/CD133+ cells. a) Morphology of A549 and A549/CD133+ cells. b) Hsp90 expression levels were assessed in A549/CD133+ cells by cellular fractionation and Western blotting analysis. c) Immunofluorescence analysis of cell surface Hsp90 in A549/CD133+ cells. d) Immunofluorescence assays of FITC-labeled P7 binding to A549/CD133+ cells. Competition of P7 binding to A549/CD133+ cells by excess free P7. [file 13045_2022_1274_MOESM5_ESM.pdf]

Supplemental Material

DTX-P7, a peptide-drug conjugate, is highly effective for non-small cell lung cancer

P7 Targeting of A549/CD133^{+} cells via Hsp90

Cytotoxic agents that target killing rapidly proliferating cells are often difficult to produce good curative effects in the dormant or quiescent CSCs in tumor tissues (34, 35). As a member of the transmembrane glycoprotein family, CD133 (also known as AC133 and prominin-1) was originally used as a surface antigen specific to human hematopoietic stem cells (36). In our previous study, CD133 was identified as a biomarker of CSLCs in NSCLC as evidenced by the fact that CD133^{+} cells isolated from A549 cells showed dramatic migration and invasion potential (37). A549/CD133^{+} cells and A549 cells showed significant difference in growth morphology (supplemental Fig. S4a). Like most stem cells, A549/CD133^{+} cells grew into non-adherent spheres, rather than monolayers of adherent cells. The presence of Hsp90 protein on the membrane of A549/CD133^{+} cells was also confirmed by Western blotting and immunofluorescence staining (supplemental Fig. S4b-c). In addition, by using a fluorescein isothiocyanate (FITC)-labeled P7, we identified the binding potential of P7 to A549/CD133^{+} cells while competition of A549/CD133^{+} cells to FITC-P7 by pretreatment with excess unlabeled P7 further verified the potential use of P7 for treatments of A549/CD133^{+} cells (supplemental Fig. S4d).

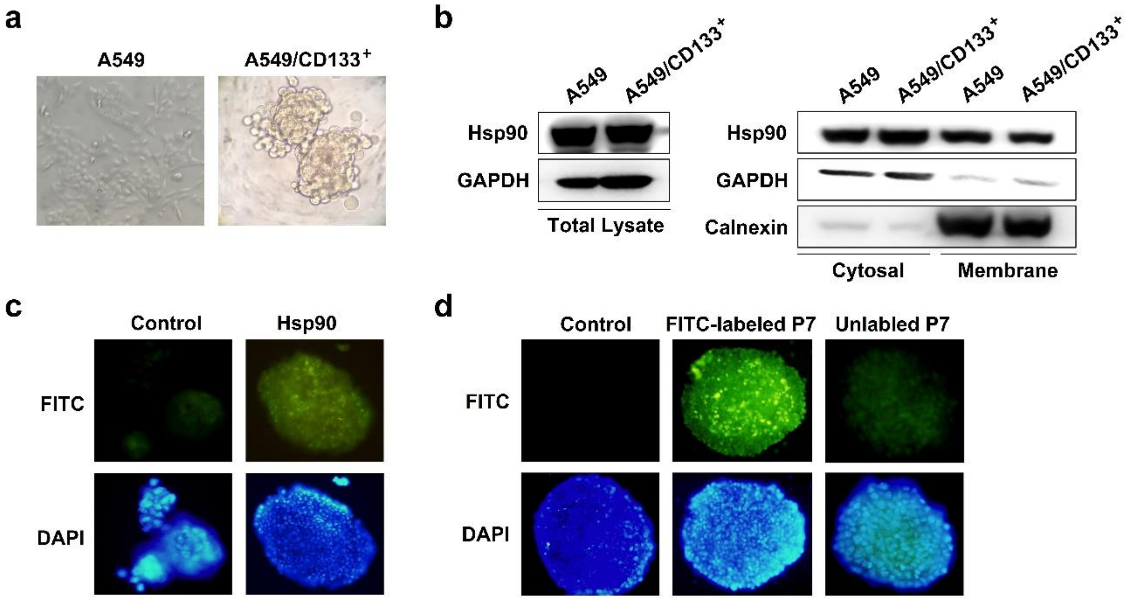

Fig.S4. Cell growth morphology of cancer stem cell-like A549/CD133$^+$ cells and identification of cell surface Hsp90 in A549/CD133$^+$ cells. a) Morphology of A549 and A549/CD133$^+$ cells. **b)** Hsp90 expression levels were assessed in A549/CD133$^+$ cells by cellular fractionation and Western blotting analysis. **c)** Immunofluorescence analysis of cell surface Hsp90 in A549/CD133$^+$ cells. **d)** Immunofluorescence assays of FITC-labeled P7 binding to A549/CD133$^+$ cells. Competition of P7 binding to A549/CD133$^+$ cells by excess free P7.
