# Supplementary material for: DTX-P7, a peptide–drug conjugate, is highly effective for non-small cell lung cancer
Source: J Hematol Oncol. 2022 Jun 3;15:73. doi: 10.1186/s13045-022-01274-8 (PMC9164557; doi:10.1186/s13045-022-01274-8)
Supplement: Supplementary file 6 — Additional file 6: Supplementary Figure S5. Effects of DTX-P7 on survival of A549/CD133+ cells and PKH26 staining of A549/CD133+ cells. a) Cell viability of DTX-P7 and DTX in A549/CD133+ cells following 48-h treatment. b) A549/CD133+ cells were stained by PKH26 followed by fluorescence detection at Day 0, 1, 6 and 10. c) Representative fluorescence-activated cell sorting profile of A549/CD133+ cells selected for sorting 10 days after PKH26 staining as compared with those of the unstained control (negative control) and Day 0. [file 13045_2022_1274_MOESM6_ESM.pdf]

Supplemental Material

DTX-P7, a peptide-drug conjugate, is highly effective for non-small cell lung cancer

Effect of DTX-P7 on survival of A549/CD133$^+$ cells

For this, we performed a cell viability assay when A549/CD133$^+$ cells were exposed to DTX-P7. As shown in supplemental Fig. S5a, DTX-P7 showed mild suppression on survival of A549/CD133$^+$ cells. Additionally, to determine the effects of DTX-P7 on cancer stem cell survival, A549/CD133$^+$ cells were stained with PKH26, a lipophilic dye which declines with every round of division and distinguishes rapidly and slowly proliferating cells by fluorescence intensity in cells (supplemental Fig. S5b). 10 days after PKH26 staining, A549/CD133$^+$ cells were divided into PKH^{low} and PKH^{high} populations based on dye retention (supplemental Fig. S5c). Following treatments with DTX-P7 or DTX for 72 h, both quiescent/slowly proliferating PKH^{high} cells and fast proliferating PKH^{low} cells were more sensitive to DTX-P7 than DTX (Fig. 2c in the main text).

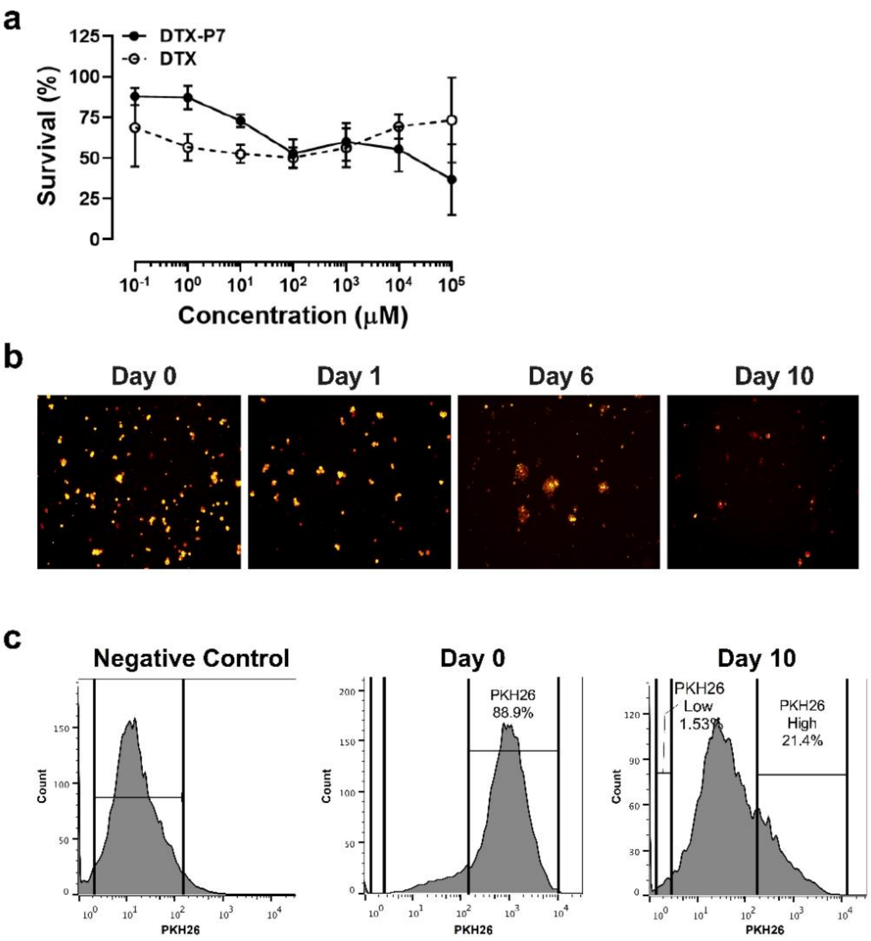

Fig.S5. Effects of DTX-P7 on survival of A549/CD133$^+$ cells and PKH26 staining of A549/CD133$^+$ cells. a) Cell viability of DTX-P7 and DTX in A549/CD133$^+$ cells following 48-h treatment. **b)** A549/CD133$^+$ cells were stained by PKH26 followed by fluorescence detection at Day 0, 1, 6 and 10. **c)** Representative fluorescence-activated cell sorting profile of A549/CD133$^+$ cells selected for sorting 10 days after PKH26 staining as compared with those of the unstained control (negative control) and Day 0.
